# EvoMBN: Evolving Multi-Branch Networks on Myocardial Infarction Diagnosis Using 12-Lead Electrocardiograms

**DOI:** 10.3390/bios12010015

**Published:** 2021-12-29

**Authors:** Wenhan Liu, Jiewei Ji, Sheng Chang, Hao Wang, Jin He, Qijun Huang

**Affiliations:** School of Physics and Technology, Wuhan University, Wuhan 430072, China; WHliu@whu.edu.cn (W.L.); jjw_whu@163.com (J.J.); changsheng@whu.edu.cn (S.C.); wanghao@whu.edu.cn (H.W.); jin.he@whu.edu.cn (J.H.)

**Keywords:** myocardial infarction (MI), genetic algorithm (GA), electrocardiogram (ECG), convolutional neural networks (CNN), architecture optimization

## Abstract

Multi-branch Networks (MBNs) have been successfully applied to myocardial infarction (MI) diagnosis using 12-lead electrocardiograms. However, most existing MBNs share a fixed architecture. The absence of architecture optimization has become a significant obstacle to a more accurate diagnosis for these MBNs. In this paper, an evolving neural network named EvoMBN is proposed for MI diagnosis. It utilizes a genetic algorithm (GA) to automatically learn the optimal MBN architectures. A novel fixed-length encoding method is proposed to represent each architecture. In addition, the crossover, mutation, selection, and fitness evaluation of the GA are defined to ensure the architecture can be optimized through evolutional iterations. A novel Lead Squeeze and Excitation (LSE) block is designed to summarize features from all the branch networks. It consists of a fully-connected layer and an LSE mechanism that assigns weights to different leads. Five-fold inter-patient cross validation experiments on MI detection and localization are performed using the PTB diagnostic database. Moreover, the model architecture learned from the PTB database is transferred to the PTB-XL database without any changes. Compared with existing studies, our EvoMBN shows superior generalization and the efficiency of its flexible architecture is suitable for auxiliary MI diagnosis in real-world.

## 1. Introduction

**Figure 1:**
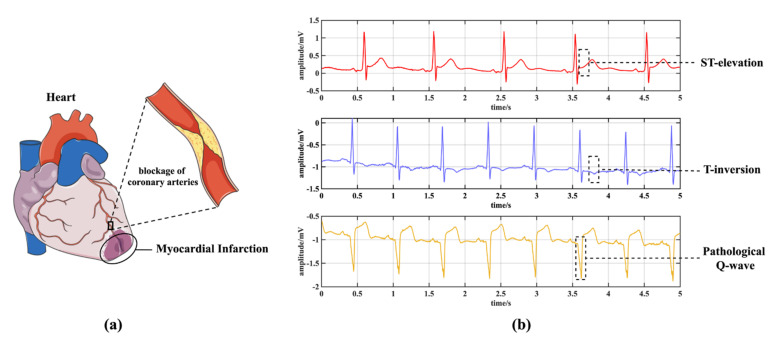
An introduction for MI. (**a**) The causes of MI. (**b**) The typical waveforms of MI.

Nowadays, cardiovascular disease (CVD) has become one of the leading causes of death around the world, especially in developing countries [1]. Considering the detailed categories of CVDs, myocardial infarction (MI, or heart attack) is known to be a higher risk of morbidity and mortality, accounting for 15 million deaths every year [2]. As shown in Figure 1a, MI is mainly caused by a blockage of the coronary arteries that cuts off the blood supply to the heart. The reduction of oxygen and nutrients may result in life-threatening damage to the myocardium, followed by an irreversible necrosis if not treated promptly [3]. Therefore, early MI diagnosis is crucial for patients to improve prognosis. Electrocardiogram (ECG) is widely used in MI diagnosis because it is non-invasive and convenient [4]. It usually consists of twelve leads, including three standard limb leads (I, II, III), three augmented limb leads (aVR, aVL, aVF), and six precordial leads (V1~V6). As shown in Figure 1b, MIs can manifest as abnormal waveforms in ECG signals, such as pathological Q-waves, ST elevations, T inversions, and so on [3,5]. Note that, MI can be categorized into several types based on location, corresponding to the aforementioned abnormal waveforms from specific leads, respectively. For instance, to detect anterior myocardial infarction (AMI), lead I, aVL, V5, and V6 deserve more analysis [6]. As for inferior myocardial infarction (IMI), the most significant leads are II, III, and aVF [6]. Cardiologists diagnose MIs by examining all the signals from the 12 leads, which is a tedious and time-consuming process. Thus, automated MI diagnosis algorithms are proposed and deployed to assist cardiologists.

For the conventional MI diagnosis algorithms using ECGs, statistical machine learning is adopted to distinguish MIs from normal types or other CVDs. It requires complex feature-engineering and classifier selection. In existing studies, waveform features (QRS-duration, QRS-amplitude, ST-segment level, T-amplitude, and so on) [7,8,9], transform features (coefficients of wavelet transform, discrete cosine transform, singular value decomposition, and so on) [10,11,12,13], and statistical features (entropy-based features, sub-band energy features, and so on) [14,15,16] are often employed to represent individuals. For classifier selections, Support Vector Machines (SVM) [12,13,15], K-Nearest Neighbors (KNN) [9,12,14,15], Decision Trees (DT) [8,12,16], and Random Forests (RF) [12] have demonstrated good performances. Obviously, feature-engineering requires much medical expertise, and the performances of these algorithms depend on the quality of the hand-crafted features. To overcome these limitations, Deep Learning (DL) models are introduced to the ECG-based MI diagnosis, which can learn critical features from data without manual intervention [17]. The most commonly used models are Convolutional Neural Networks (CNNs), Recurrent Neural Networks (RNN), and their variants. Particularly, 1-D CNNs were used in [18] to detect MIs using lead II. It achieved an accuracy of 95.22% without feature extraction and selection. In [19], a multi-layer Long Short-Term Memory (LSTM) network (a typical variant of RNN) was employed to analyze single-lead ECGs and identify MI patients. This model was tested on two different ECG databases, and the accuracies were 77.12% and 84.17%, respectively. Similar LSTM models for MI diagnosis were also developed and evaluated in [20]. For MI diagnosis on wearable devices, a lightweight Binary CNN (BCNN) was designed in [21]. All the parameters of BCNN are represented in binary, which can dramatically save the computational resources. In addition, an acceptable result (accuracy = 91.22%) was achieved by the model in MI detection using single-lead ECGs. To explore more leads, signals from lead II, III, and aVF were fed into a shallow CNN model to diagnose IMIs in [22]. An accuracy of 84.54% was obtained in subject-oriented experiments. An ML-CNN proposed in [23] is an impressive variant of standard CNN. For generalized anterior myocardial infarction (GAMI) detection, it utilized lead V2, V3, V5 and aVL to analyze and achieve an accuracy of 96.00%. Based on the same leads, an ML-Net was also developed in [24] for GAMI detection. Although the models using single lead or multiple leads (<12 leads) can produce accurate MI detection according to the experiment results, limited lead information may prevent these models from extending to a more complex application in the real-world [25]. Using all of the 12 leads, MFB-CNN, MFB-CBRNN, ML-ResNet, and MFB-LANN were proposed in [26,27,28,29], respectively. The MI detection accuracies were all greater than 93% in these three studies. Additionally, 12-lead ECGs can also be transferred to 2-D images and can be processed by existing deep networks of computer vision [30,31,32], but the rationality of these approaches may require more exploration since the ECG images are different from the natural 2-D images [33]. Compared with the conventional approaches, the DL-based algorithms have shown great advantages because of better generalization and robustness, which has gained increasing attention in the past few years. 

**Figure 2:**
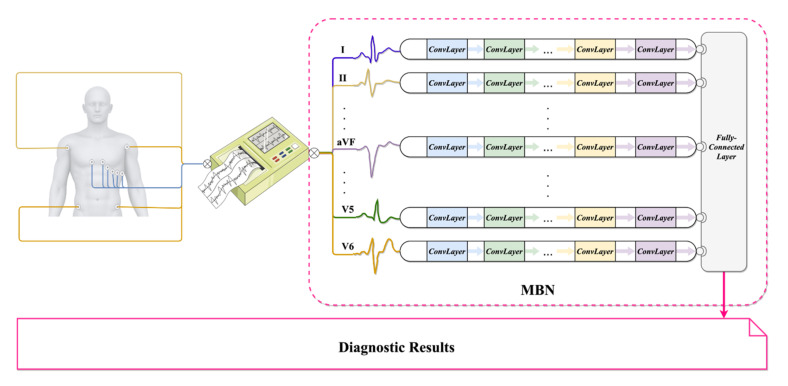
The conventional MBN skeleton. ***ConvLayer***: A convolutional layer or a pooling layer.

In fact, the aforementioned MFB-CNN, MFB-CBRNN, ML-ResNet, ML-Net, and MFB-LANN employ the same Multiple-Branch Network (MBN) skeleton as depicted in Figure 2. Each lead has its own CNN-based branch network for feature learning. A global fully-connected layer summarizes features from all of the leads and produces final results. Unlike normal DL models, the MBN skeleton is specially designed for multi-lead processing to exploit the diversity and integrity of 12-lead ECGs [26]. However, a fixed architecture is used for all of the branch networks, which may not be the best one for each lead. It limits the flexibility of the whole model, whereas manual architecture optimization is always a difficult task [17]. A genetic algorithm (GA) is a typical heuristic optimization algorithm that does not require much domain knowledge [34]. It mimics the biological evolution by performing crossover, mutation, selection, and fitness evaluation in an iterative manner. A GA and its variants have been successfully applied to Neural Architecture Search (NAS), a technique that can automatically design optimal architectures of neural networks [35]. For example, an EvoCNN for image classification was developed in [36] using GAs without manual tuning. Moreover, similar automatically designed CNNs were proposed in [37,38]. Compared with the manually designed architectures, these automatic models have shown significant advantages in terms of classification accuracy and the number of parameters. Unfortunately, the above GA-optimized models are only suitable for 2-D image classification using standard 2-D CNNs, which cannot be directly applied to ECG-based MI diagnosis using the MBN skeleton. Thus, evolving the MBN skeleton automatically through GAs is a critical problem for a more accurate and flexible MI diagnosis using 12-lead ECGs. 

In this paper, an evolutional MBN (EvoMBN) is proposed to model the 12-lead ECGs for MI detection and localization. In particular, it combines the GA-based NAS technique and the MBN skeleton to automatically learn an optimized architecture. The MBN skeleton ensures that it remains suitable for multi-lead ECG processing, and the automatic GA optimization enhances its flexibility to achieve a better generalization. Furthermore, it requires no hand-designed features since it is a DL model. In detail, the main contributions of this paper are as follows:

(1) To balance computational burden and algorithm flexibility, the EvoMBN employs a GA to implement a constrained architecture optimization based on the MBN skeleton. Specifically, a limited number of branch net layers are given in advance. Then GA iterations are performed to automatically learn an optimal depth for each branch net. An efficient architecture encoding strategy is proposed to represent the whole model, making it possible to globally search the optimal solution.
(2) To efficiently summarize all the leads and produce final results, a novel Lead Squeeze and Excitation (LSE) block that consists of a fully-connected layer and an LSE mechanism is established. The LSE extends the typical SE [39] to weight leads which are more relevant to the target categories. Compared with a simple fully-connected layer for feature summary, the LSE block can achieve a better performance in our experiments.
(3) To comprehensively evaluate the generalization of EvoMBN, five-fold cross validation is performed on the Physikalisch-Technische Bundesanstalt (PTB) diagnostic ECG database [40] under the inter-patient paradigm [41]. The inter-patient paradigm is a more practical evaluation method, as it considers the model generalization on unseen patients. Furthermore, the best EvoMBN architecture learned from the PTB database is directly transferred to the MI detection and localization on the PTB-XL database [42], a larger ECG database which shares no records with the PTB database. To the best of our knowledge, there has not been any architecture transfer developed for cross-database evaluations in ECG-based MI diagnosis. Finally, the superior results in the experiments demonstrate the robustness of our model.

The rest of this paper is organized as follows. The datasets used in the model development and the details of our model are introduced in Section 2. Section 3 shows the experimental design and results. A comprehensive discussion is provided in Section 4. Finally, Section 5 concludes the whole paper.

## 2. Materials and Methods

**Figure 3:**
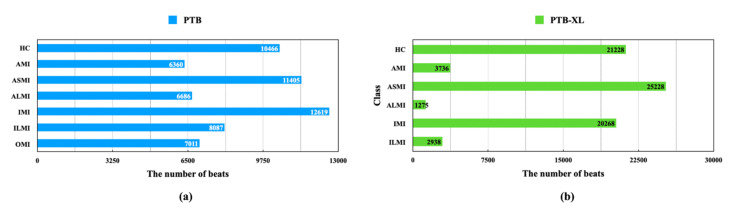
The statistic information of the 2 used databases. (**a**) PTB (**b**) PTB-XL.

First, this section introduces the ECG datasets used for MI diagnosis, including the PTB database and the PTB-XL database. In addition, it presents the preprocessing method used for the ECG signals and the statistical information of the categories considered in MI detection/localization. Particularly, the PTB-XL database is employed to evaluate the automatically learned architecture transferred from the PTB database. Figure 3 shows the statistical information of these 2 databases. 

**Figure 4:**
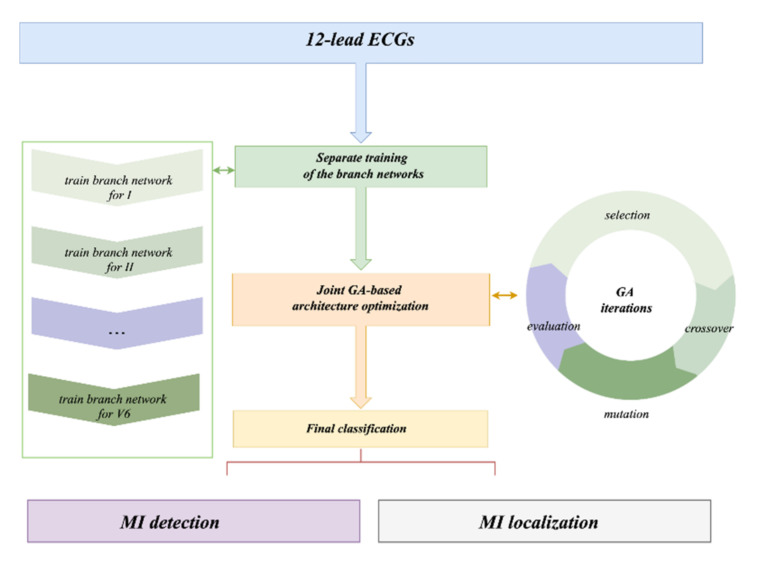
A flowchart of the proposed method.

Moreover, the EvoMBN for MI diagnosis using 12-lead ECGs is elaborately described in this section. It consists of 3 main phases: separate training of the branch networks, joint GA-based architecture optimization, and final MI detection/localization. As a subnet for branch summary, the LSE block is used in both the architecture optimization and final classification. In addition, a flowchart of the proposed method is shown in Figure 4.

### 2.1. Datasets

#### 2.1.1. The PTB Database

The PTB diagnostic database is the most commonly used ECG database in studies related to MI diagnosis algorithms. According to [40], it contains 549 12-lead records sampled at 1000 Hz from 290 patients. A diagnostic result summarized by several cardiologists is attached to each record. As for MI detection/localization, 368 MI records from 148 patients and 80 healthy control (HC) records from 52 patients can be involved in the algorithm research. In detail, there are 6 location-based MI subcategories with sufficient records in the database, including anterior MI (AMI), antero-septal MI (ASMI), antero-lateral MI (ALMI), inferior MI (IMI), infero-lateral MI (ILMI), and other MI (OMI). Note that, the OMI is a collective term for several MI subcategories with insufficient records [43]. Therefore, the MI detection is a binary classification that distinguishes MIs from HCs, while MI localization is a multi-class classification that should determine the detailed MI subcategories. 

In order to achieve a trade-off between computational burden and information retention, all the ECG signals were downsampled to 250 Hz as in the existing studies [23,27,29]. In addition, Daubechies 6 wavelet filtering [44] was adopted to remove noise and baseline wander in the ECG signals. In particular, our algorithm is developed on ECG heartbeats (or beat for short). A heartbeat is a P-QRS-T cycle, which is a basic unit of ECG [4]. To segment beats from a whole record, a NeuroKit2 R-wave detection algorithm was employed [45]. Once an R-wave was detected, a segment that includes 127 samples to the left and 128 samples to the right of an R-wave position was selected as an ECG beat of 256 samples (127 + 128 + 1 = 256). The reason for setting the length to 256 was that it is more suitable for the processing of CNN models [46]. Furthermore, each beat was normalized by z-score to remove baseline offset and amplitude scaling, which can be formulated as:

(1) $$z=\left(x-\mu \right)/\delta$$

where *x* is an ECG signal, and *µ* and 𝛿 denote the mean value and the standard deviation of the signal, respectively. Moreover, the statistical information based on categories is shown in Figure 3a.

#### 2.1.2. The PTB-XL Database

The PTB-XL is another open-source 12-lead ECG database used in this research; it was established by the same institution as the PTB diagnostic database [42]. It provides 21,837 12-lead records of 10 s from 18,885 patients and shares no records with the PTB database. The sampling rate of the ECG signals is 500 Hz or 100 Hz, corresponding to 2 versions. The version sampled at 500 Hz was selected and downsampled to 250 Hz in this study. The other preprocessing steps were similar to those applied to the PTB database. Note that, the aforementioned OMI is not a specific MI subcategory. The actual MI subcategories included by the OMI in the PTB-XL database are different from those in the PTB database. Therefore, OMI is excluded here. The statistical information of the data used for MI diagnosis is illustrated in Figure 3b.

### 2.2. Separate Training of the Branch Networks

**Figure 5:**
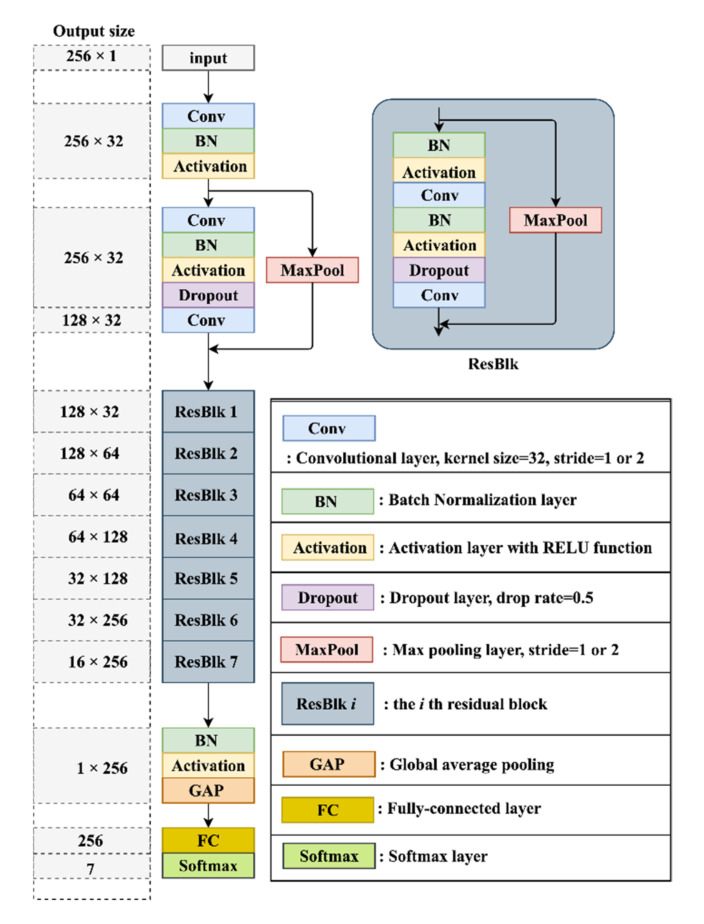
The detailed architecture of a branch network.

For the MBN skeleton, the role of the branch networks is to learn the critical features of each lead. Unlike the conventional MBNs [26,27,28,29] that synchronously train all the branch networks, a separate scheme was utilized here. It makes the model more flexible and reusable since the multi-layer features learned by different branch networks can arbitrarily combine without any extra training. The architecture of each branch network was developed based on the efficient Residual Network (ResNet) proposed in [46]. Particularly, a residual architecture with 17 convolutional layers was designed, as described in Figure 5. To make the branch networks more sensitive to detailed features, each branch network was trained to classify the 6 MI subcategories (AMI, ASMI, ALMI, IMI, ILMI, OMI) and HC. Note that, this multi-class classification is not the final MI localization, it is just a strategy for the feature learning of the branch networks. 

To train the branch networks, weighted cross entropy loss was employed, which can alleviate the effects of the class imbalance in the PTB or the PTB-XL database. It can be computed as:

(2) $$Loss=\sum_{i}^{c}\omega_{i}y_{i}log\left(p_{i}\right)+\left(1-y_{i}\right)log\left(1-p_{i}\right)$$

where *c* is the number of classes considered in the training, ω*_i_* is the weight of the class *i*, *y_i_* and *p_i_* denote the desired and actual output, respectively. Generally, larger weights should be assigned to classes that have fewer samples, making the network pay more attention to these classes. To this end, a weighting scheme inspired by [47] was utilized to balance the multi-class losses. Moreover, the loss was minimized using the Stochastic Gradient Descent (SGD) with momentum. The initial learning rate was set to 0.1 and decreased by a factor of 10 every 10 epochs. The momentum factor was 0.9 and the batch size was 128. Each branch network was trained for 30 epochs. 

Finally, 12 branch networks were obtained as feature extractors. Hierarchical features can be generated by the multi-layer architecture of the branch networks [17]. However, conventional MBN models only exploit the top-level features from the tails of all the branch networks. This homogeneous level combination may not be optimal for all the leads since each lead has its own particular pathological information [6]. Therefore, the optimal feature level, corresponding to the features from the optimal depth of the branch networks, should be explored to implement a more accurate MI diagnosis.

### 2.3. LSE Block

**Figure 6:**
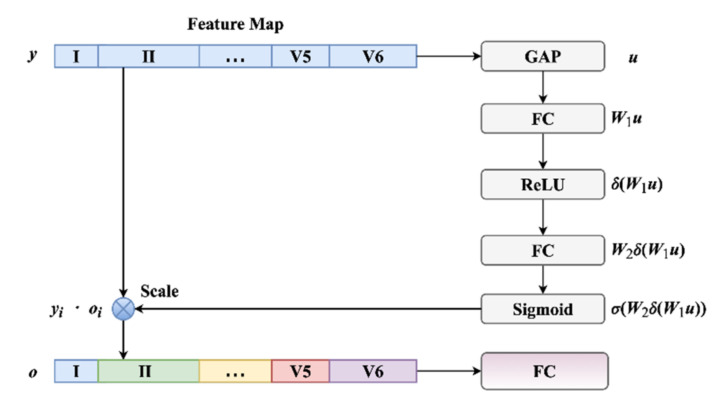
The operations of an LSE block.

Unlike the conventional MBN skeleton, a novel LSE block was employed to summarize all the features from the branch networks, which consists of a fully-connected layer and an LSE mechanism. The standard SE is designed to explicitly model a channel-wise feature importance in a specific layer [39], whereas our LSE transfers the standard SE to a lead-wise version. Figure 6 illustrates the LSE block in detail. Note that, the features from each lead were preprocessed by a Global Average Pooling (GAP) layer before being fed into our LSE block. In addition, GAP was proposed to squeeze the multi-lead information from all the branch networks. After that, Let *u_i_* was the squeezed feature from lead *i* and ***u*** = [*u*_1_, *u*_2_, …, *u*_12_]^T^ which concatenated all these features, the excitation values can be computed by 2 fully-connected layer as:

(3) $$e=\sigma \left(W_{2}\gamma \left(W_{1}u\right)\right)$$

where 𝜎 and 𝛾 denote the sigmoid and the Rectified Linear Unit (ReLU) function, respectively. ***W***_1_ ∈ ℝ^(12/*r*)×12^ is the weight of the first layer and ***W***_2_ ∈ ℝ^12×(12/*r*)^ is the weight of the second layer. Reduction factor *r* was set to 1 here. The excitation vector ***e*** = [*e*_1_, *e*_2_, …, *e*_12_] was applied to scale the features from multiple leads as:

(4) $$o_{i}=e_{i}\cdot y_{i}$$

where ***o****_i_* is the final output feature vector of lead *i*, *y_i_* is the input feature vector of lead *i*. In addition, a fully-connected layer was employed to perform the final classification. Figure 6 illustrates the LSE block in detail. In short, the LSE block can help the model discover critical features from relevant leads. 

**Figure 7:**
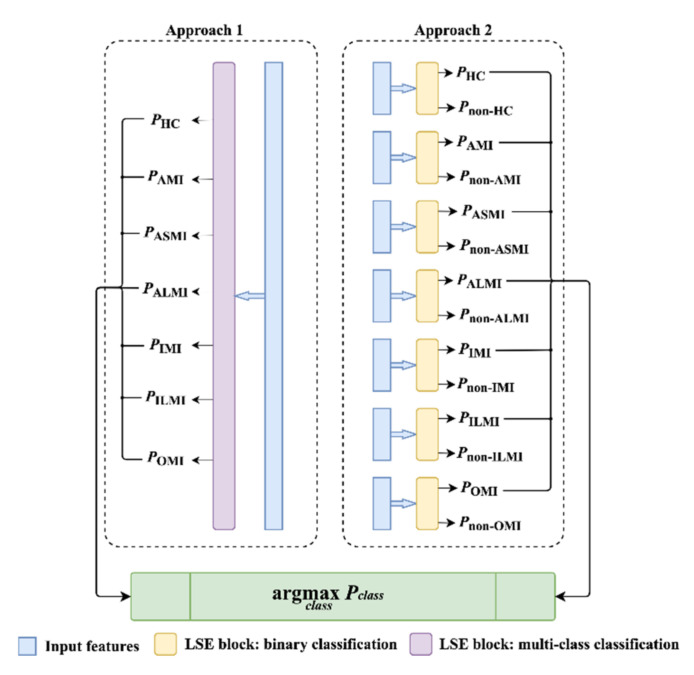
The implemental approaches for MI localization.

As for the MI diagnosis in this paper, the LSE block can implement the MI detection by performing a binary classification. However, there are 2 approaches that can implement the MI localization. As shown in Figure 7, MI localization can be regarded as a plain multi-class classification. In addition, it can be transformed to a group of binary classification. Each element in the group is used to distinguish a specific category (positive) from the other categories (negative). The category with the maximum positive probability is the final output category. These 2 approaches are both evaluated and analyzed in the following sections. Moreover, the LSE block was trained for 30 epochs using Adam optimizer [48] to minimize the weighted cross entropy loss, as introduced in Section 2.2. 

### 2.4. Joint GA-Based Architecture Optimization

#### 2.4.1. Encoding Strategy and Problem Formulation

**Figure 8:**
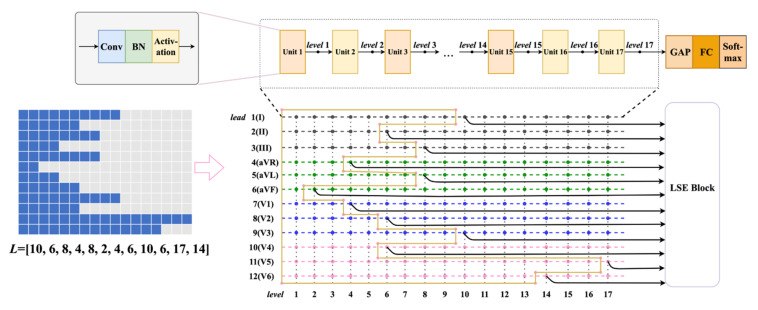
The diagram of the proposed EvoMBN.

To automatically discover the optimal feature levels, a GA was adopted to optimize the conventional MBN skeleton. Generally, a level combination can be formulated as ***L*** = [*l*_1_, *l*_2_, …*l_i_*, …, *l*_12_], *l_i_* denotes the feature level of lead *i*. Once the feature level of a lead is given, the depth of the corresponding branch network is determined. Therefore, the ***L*** can encode the architecture of the whole model, the GA optimization is to discover the optimal ***L*** in a specific search space. According to the conventions of CNN models, a basic unit usually consists of a convolutional layer, a Batch Normalization (BN) layer, and an activation layer, regardless of additional residual connections. Thus, each proposed branch network stacks up 17 basic units, which can be treated as 17 feature levels. As shown in Figure 8, an index ranging from 1 to 17 was assigned to each level. Note that, only the levels with even indices were considered in the optimization. The reasons for this level limitation can be summarized in 2 aspects. First, it can simplify the task and alleviate the computational burden. Second, features from adjacent levels may be similar and redundant [49]. The level limitation can reduce the information redundancy and enhance the robustness of the algorithm. Moreover, the features from the final GAP layer are usually critical for the final classification [26,27,28,29], which correspond to the top level (17th level) of the branch network. Thus, the top level was also considered in the optimization. 

Finally, the 12-lead features from the levels represented by ***L*** were employed to train an LSE block for MI detection/localization. An example (***L*** = [2, 4, 4, 6, 6, 6, 8, 8, 10, 10, 14, 17]) is given in Figure 8. In summary, the GA-based automatic optimization can be formulated as:

(5) $$\left\{\begin{array}{c} \underset{L}{\mathrm{argmax}}f\left(A\left(L\right)\right)\\ s.t.\;\left|L\right|=12,\\ l_{i}\leq 17,\;i=1,2,\ldots ,12\\ l_{i}\;mod\;2=0\;or\;l_{i}=17 \end{array}\right.$$

where function *A* (•) is to decode the ***L*** to a specific architecture, and function *f* (•) evaluates the fitness of the architecture. The search space is defined by the 3 constraints. The |***L***| denotes the length of ***L***. In theory, this problem can be solved by enumerating all the possible values of ***L***, but it cannot obtain a good result within the acceptable time [50]. In contrast, GAs can implement a more efficient search by performing evolutional iterations that consist of selection, crossover, mutation, and fitness evaluation. It is expected to obtain superior results after several generations. The detailed operations of the GA are introduced in the following sections. 

#### 2.4.2. Initialization

As a population-based algorithm, a GA usually starts with a set of randomized individuals. In this study, a base population was randomly initialized via uniform distribution sampling. Each individual was represented by an L that corresponds to a special architecture of MBN. The size of the population was set to 100 here. After that, a fitness value was computed for each individual, and the method used is elaborately described in the next section.

#### 2.4.3. Fitness Evaluation

For GA optimization, a fitness value indicates the quality of an individual in the population. Particularly, there were 2 phases for fitness evaluation in this study. First, the architectures represented by the individuals (denoted by ***L***) were set up. Multi-level features are extracted from the branch networks to train an LSE block. Second, the fitness value of an ***L*** was calculated as:

(6) $$fitness=\alpha \cdot F1+\beta \cdot Accuracy-\eta \cdot \sum_{i}^{12}l_{i}$$

where *F*1 and Accuracy denote the f1 score and the classification accuracy of the model represented by the ***L***, respectively. In addition, *l_i_* is the *i*th element of the ***L***, and the summation of all the elements can indicate the complexity of the model. Parameter *𝛼*, *β*, and *η* are the weights (>0) to balance the factors. The GA aims to discover the individual with the maximum fitness value, which corresponds to a lightweight model with a high f1 score and accuracy here. To assign the priorities, *𝛼*, *β*, and *η* were set to 1, 1 × 10^−1^, and 1 × 10^−5^, respectively. In other words, model performance is more significant for the GA than model complexity since our basic target is to implement a more accurate MI diagnosis. To discover the best individual in a group of models with similar performances, the most lightweight one is preferred since it can reduce the computational burden. Fitness evaluation is the fundamental step for the GA selection, as illustrated in the following part.

#### 2.4.4. Selection

The selection process is designed to obtain the best individuals used to produce the next generation. Based on the fitness value, all the individuals were sorted in descending order. Then the first 10 individuals were selected as the parents to generate offspring. This means that the individuals with higher fitness values are always selected. Finally, the parents and the new offspring constitute the next generation of the population. The essential operations to generate offspring are crossover and mutation, which are shown in Section 2.4.5.

#### 2.4.5. Crossover and Mutation

**Figure 9:**
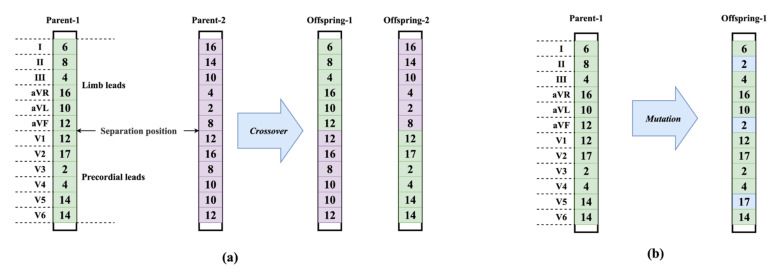
(**a**) An example of crossover. (**b**) An example of mutation.

To generate new offspring, crossover and mutation are performed on the parent individuals. For the crossover operation, 2 parent individuals were randomly selected at first. Then a one-point crossover scheme was performed since it is widely used in the GA-based optimization [51]. The separation position is the central point of an individual. As a result, 2 new individuals were generated from the 2 parent individuals. An example of the proposed crossover is depicted in Figure 9a. Specifically, the proposed crossover operation can exactly exchange the architecture information of limb leads or precordial leads. It preserves the completeness of the information from the 2 critical groups (limb and precordial leads). Thus, it is more suitable for the 12-lead system than the version based on random separation position. 

Unlike the crossover using 2 parent individuals, mutation can produce offspring with only 1 parent individual. Given an individual ***L*** having 12 elements *l*_1_~*l*_12_, the mutation randomly selects *k* elements and resets each one to 2 with the probability *p*_1_ or 17 with the probability *p*_2_. Note that, the mutation operator was only performed on a portion of all the individuals. The mutated individuals were randomly selected with the probability *p_m_*. In detail, *p*_1_, *p*_2_, and *p_m_* were set to 0.8, 0.2, and 0.25, respectively. The number of mutated elements *k* was set to 3. Figure 9b shows an example of the mutation.

#### 2.4.6. Iteration

The selection, crossover, mutation, and fitness evaluation constitute a GA iteration. Multiple iterations were performed to promote the fitness of the whole population. After that, the individual with the maximum fitness value is regarded as the best feasible solution to problem (5). The upper bound of the iterations was set to 10 here. Moreover, an early stop strategy was proposed to save computational time. Once the maximum fitness value of the population has not changed for 2 generations, the GA iterations should be stopped.

## 3. Results

This section illustrates the experimental design and results of the MI detection and localization on the PTB database. There are two commonly used paradigms for performance evaluation: intra-patient and inter-patient. In particular, the inter-patient paradigm splits the training and the testing dataset according to the patients. In other words, no patient overlaps exist between the training and the testing dataset. However, beats from the same patient can be included in both the training and the testing set under the intra-patient paradigm. Therefore, inter-patient is more practical than intra-patient for performance evaluation. In this study, all the experiments were performed under the inter-patient paradigm. The networks were implemented using Keras with a TensorFlow backend. 

### 3.1. MI Detection

As mentioned in Section 2.3, MI detection is a binary classification task, which distinguishes MI from HC samples. Thus, accuracy (*Acc*), sensitivity (*Sen*), specificity (*Spe*), positive predicted value (*Ppv*), and *F*1 score were used to measure the performance of MI detection. As formulated in (7)~(11), these metrics are defined by 4 parameters: True Positive (*TP*), True Negative (*TN*), False Positive (*FP*), and False Negative (*FN*). *TP* denotes the number of correctly classified positive samples, and *TN* is the number of correctly classified negative samples. The number of negative samples categorized as positive ones is defined as *FP*, whereas positive samples categorized as negative ones are *FN* samples. In MI detection, MI and HC beats are treated as positive and negative samples, respectively.

(7) $$Sen=\frac{TP}{TP+FN}\times 100\%$$

(8) $$Spe=\frac{TN}{TN+FP}\times 100\%$$

(9) $$Ppv=\frac{TP}{TP+FP}\times 100\%$$

(10) $$Acc=\frac{TP+TN}{TP+TN+FP+FN}\times 100\%$$

(11) $$F1=\frac{2\cdot TP}{2\cdot TP+TN+FP+FN}=\frac{2\cdot Sen\cdot Ppv}{Sen+Ppv}$$

**Figure 10:**
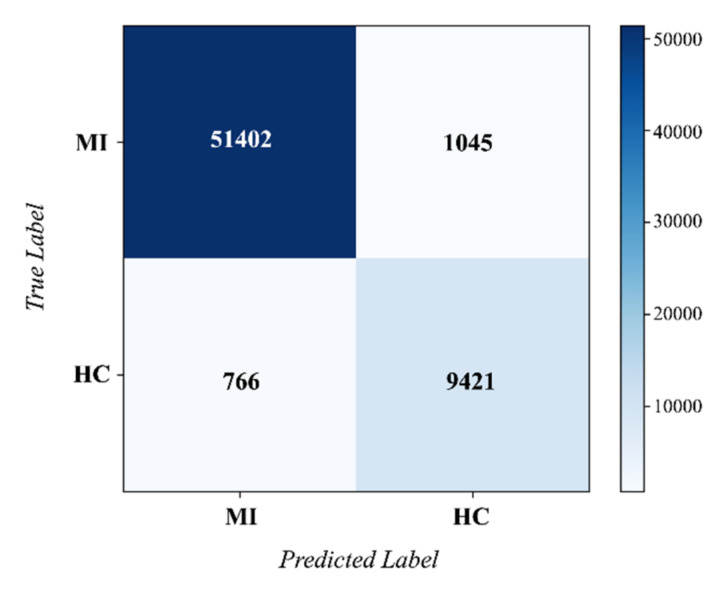
The confusion matrix of MI detection on the PTB database.

Under the inter-patient paradigm, five-fold cross validation was performed on the PTB database. Then the confusion matrix across the folds was obtained, as shown in Figure 10. The performance metrics can be calculated according to this confusion matrix. Our EvoMBN achieved an accuracy of 97.11% in MI detection. The *Sen*, *Spe*, *Ppv*, and *F*1 were 98.53%, 90.02%, 98.01%, and 0.983, respectively. Specifically, the *Spe* was a little lower than the other four metrics, which means that the model is more prone to classify HC beats as MI beats. This may be caused by the class imbalance mentioned in Section 2.2. Although weighted cross entropy is employed to alleviate the imbalance, it cannot eliminate the impact completely. 

In summary, the EvoMBN has demonstrated not only accurate, but also robust MI detection on the PTB database, which indicates the efficiency of the automatic architecture optimization. Moreover, the five-fold cross validation can avoid overfitting for a specific dataset, making the results more credible.

### 3.2. MI Localization

Compared with MI detection, MI localization is a more complex multi-class classification task. As shown in Section 2, six MI-related classes and HC are involved in the PTB database. However, most existing studies [26,28,43] used five MI subcategories in the inter-patient MI localization, including AMI, ASMI, ALMI, IMI, and ILMI. In order to compare our results with these studies, a six-class (five MI subcategories and HC) MI localization was performed in the five-fold cross-validation experiments under the inter-patient paradigm. As described in Section 2.3, the MI localization can be implemented in 2 classification manners: a single multi-class classifier and a group of binary classifiers, represented by *model_m_* and *model_b_*, respectively. Therefore, the experiments based on these two manners were performed and analyzed. 

**Figure 11:**
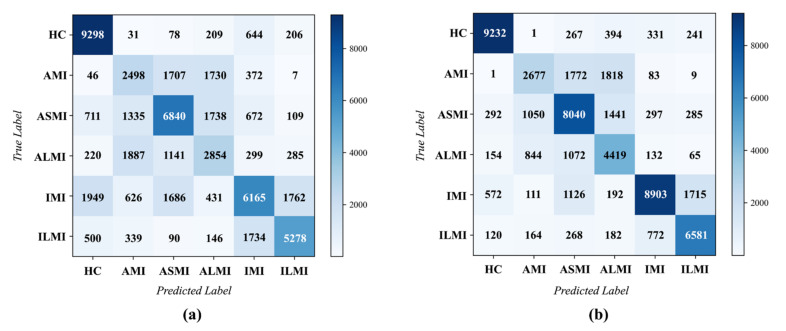
The confusion matrix of MI localization on the PTB database. (**a**) Based on a single multi-class classifier(*model_m_*). (**b**) Based on a group of binary classifiers (*model_b_*).

Figure 11a,b provides the confusion matrices across the five folds of the MI localization experiments. Furthermore, *Sen*, *Spe*, *Ppv*, *Acc*, and *F*1 were also employed to evaluate the performance of each class, as presented in Table 1 and Table 2. Obviously, *model*_b_ achieved a more accurate MI localization. In detail, the overall *Acc* was 71.65%, the average *Sen*, *Spe*, *Ppv*, and *F*1 were 69.80%, 94.34%, 69.88%, and 0.694, respectively. However, the performance of *model_m_* was not as good as that of *model_b_*. The overall Acc was only 59.21%, the average *Sen*, *Spe*, *Ppv*, and *F*1 were 57.50%, 91.81%, 56.84%, and 0.569, respectively. According to the confusion matrices, the errors were mainly caused by the misclassifications of the similar categories. For example, AMI, ASMI, and ALMI manifest as similar abnormal waveforms in ECG [6], making it prone to misclassifications. Moreover, the similarities between IMI and ILMI also resulted in the classification errors. For *model_b_*, each classifier concentrates on the critical features of a specific category. It may help the model explore the special characteristics of each MI subcategory, which can reduce the errors caused by the aforementioned similarities. 

To summarize, although MI localization is a challenging task that requires superior generalization of the algorithm, the EvoMBN obtains acceptable results based on the evolutional architectures. In addition, the experiments have demonstrated the advantages of the implement method that combines a group of binary classifiers. It is beneficial for the GA to find the best individuals since each individual can be further optimized for a specific class.

## 4. Discussion

In this section, the significant contributions of the EvoMBN are discussed based on a series of ablation experiments. Furthermore, to further verify the generalization of the algorithm, the architectures learned from the PTB database are transferred to the PTB-XL database without any changes. Moreover, a detailed comparison between the EvoMBN and the other existing methods is presented in the last part of this section.

### 4.1. The Efficiency of the LSE and GA Optimization

**Figure 12:**
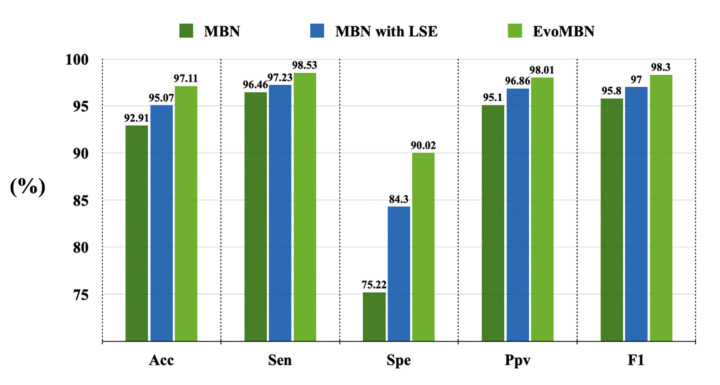
The results of the ablation experiment on MI detection.

**Figure 13:**
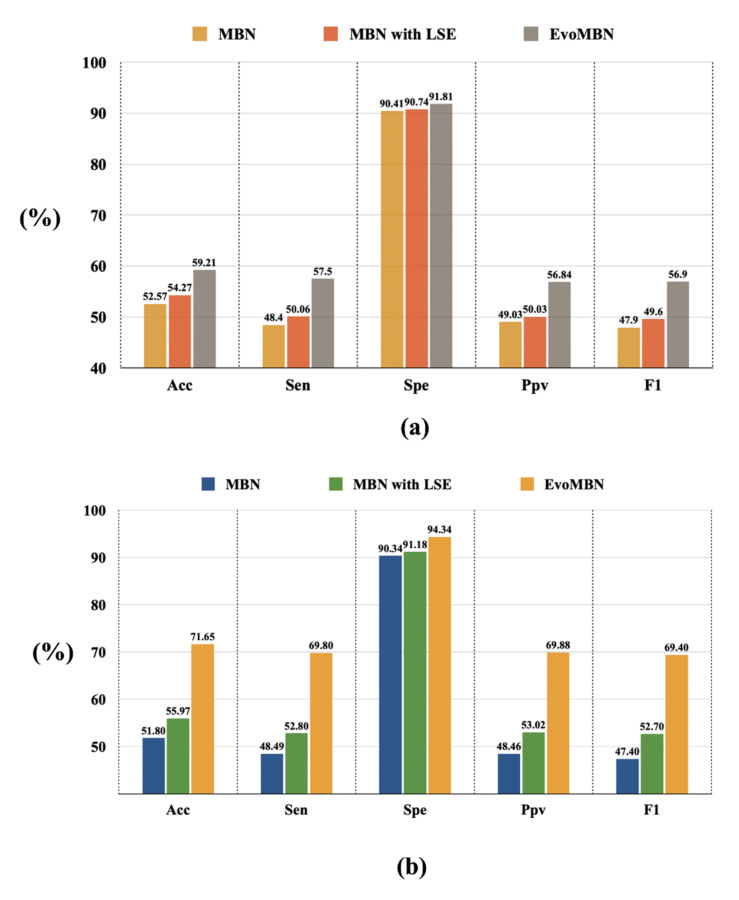
The results of the ablation experiment on MI localization. (**a**) Based on a single multi-class classifier (**b**) Based on a group of binary classifiers.

The LSE block is designed to replace the simple fully-connected layer of the conventional MBN skeleton. Then the architecture is further evolved by the GA iterations and achieves impressive performance in the experiments. The efficiency of these two strategies can be demonstrated by a series of ablation experiments. Figure 12 provides the results of the ablation experiments on MI detection. As the MI localization can be implemented by two methods, the ablation experiments using these two methods are performed. The results are shown in Figure 13a,b. Note that, all the ablation experiments are based on the inter-patient five-fold cross-validation. 

According to Figure 12 and Figure 13, the LSE block and the GA optimization can improve the model performance to some extent. The overall accuracy of MI detection increases by 4.2% with the help of these two strategies according to Figure 12. For MI localization, the improvement is more significant. As illustrated in Figure 13, the accuracy of the model based on a single multi-class classifier has risen from 52.57% to 59.21%. In addition, the model that combines a group of binary classifiers achieves an accuracy of 51.80% without the LSE block and GA optimization, whereas its accuracy increases to 71.65% with the applications of the two strategies. Therefore, the efficiency of the LSE block and GA optimization can be verified by the obvious performance improvements. 

**Figure 14:**
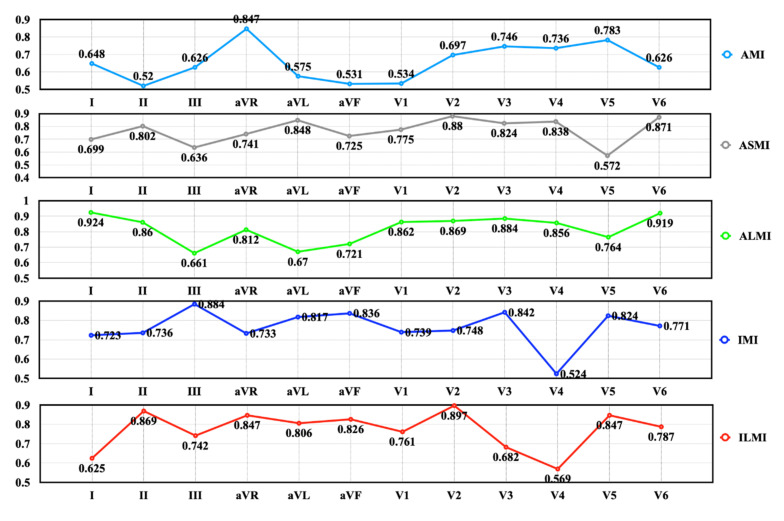
The excitation values learned by the LSE.

In particular, LSE can assign weights (excitations) to the leads, making the relevant leads more significant in the MI diagnosis. Thus, it is essential to analyze the excitation values of the 12 leads for different MI subcategories. Since the combination of binary classifiers achieves the best performance, the average lead excitation values across the five folds were computed for each MI subcategory based on these models, as presented in Figure 14. In addition, each lead corresponds to a special anatomical area of the heart [52], as illustrated in Table 3. A rough analysis was performed to check if the relevant leads are emphasized when diagnosing a specific MI subcategory. 

For AMI, V3~V5 and aVR have greater excitations, while V3 and V4 are the most relevant leads according to Table 3. Moreover, ST-segment changes in aVR are proved to be critical in the diagnosis of non-inferior MI and inferior MI [53]. Thus, aVR always has a fairly large weight (>0.7) in the MI localization, as shown in Figure 14. In the ASMI diagnosis, V2 has the largest excitation in the 12-lead system, which is associated with the septal aspect of the heart. Moreover, V3 and V4 are emphasized to a certain extent with weights greater than 0.8, corresponding to the anterior aspect. However, the LSE also assigns large weights to aVL and V6 (lateral aspect), making it more prone to misclassify ASMI as ALMI. This inference can be verified by the confusion matrix given in Figure 11. As for ALMI, the related leads include I, aVL, V5, V6, V1, and V2. Obviously, I and V6 are the most important leads for the LSE in ALMI detection according to the excitation values. Moreover, the emphasis on V3 results in the significant misclassification between ASMI and ALMI, as shown in Figure 11. In particular, II, III, and aVF are expected to have large weights in the IMI diagnosis. Actually, the LSE gives great excitation values to III and aVF. Similarly, II is regarded as one of the most critical leads for ILMI diagnosis according to the excitation values. Again, the inappropriate emphasis on V2 may lead to the considerable misclassification between ILMI and ASMI. In general, at least two relevant leads are emphasized by the LSE in the diagnosis of a specific MI subcategory, which can also indicate the efficiency of our LSE mechanism.

### 4.2. Architecture Transferring

To further evaluate the generalization of the automatically optimized model, the architectures learned from the PTB database were transferred to the MI diagnosis on the PTB-XL database. The branch networks trained on the PTB database were directly used to extract features and no additional training was performed. The architectures of the best fold in the five-fold cross validation were applied without any changes. Particularly, the implement method which is based on a combination of binary classifiers was used for MI localization, since it can achieve a better performance in the aforementioned experiments. Table 4 presents the detailed information on the transferred architecture. The LSE blocks that summarize all the features should be trained on the PTB-XL database, which can be regarded as a specific fine-tuning of the whole EvoMBN. Note that, the PTB-XL database recommends a train-test splitting method in [42] based on the inter-patient paradigm. Thus, all the experiments in this part adopted this splitting method to evaluate the models.

**Figure 15:**
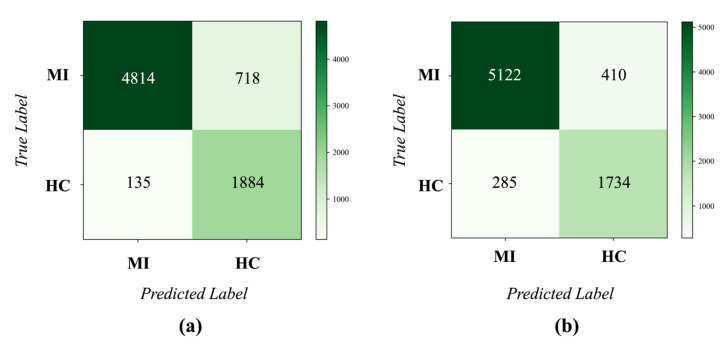
The confusion matrix of MI detection on the PTB-XL database. (**a**) MBN (**b**) EvoMBN.

**Figure 16:**
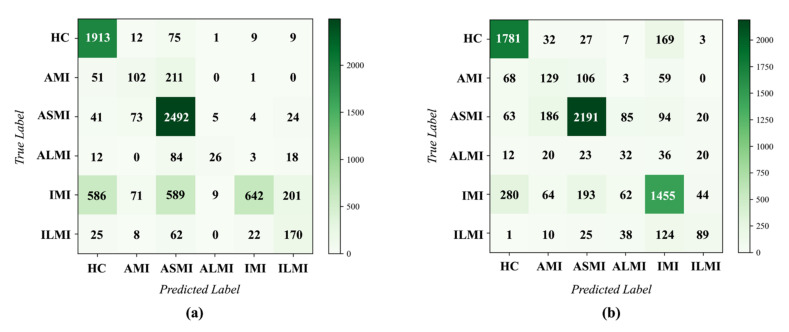
The confusion matrix of MI localization on the PTB-XL database. (**a**) MBN (**b**) EvoMBN.

To demonstrate the advantages of the EvoMBN, the model using conventional MBN skeleton was also employed to implement the MI diagnosis on the PTB-XL database. The confusion matrices are presented in Figure 15 and Figure 16, corresponding to the MI detection and localization, respectively. Moreover, *Acc*, *Sen*, *Spe*, *Ppv*, and *F*1 score were computed, as shown in Table 5 and Table 6. According to Table 5 and Table 6, the EvoMBN shows better generalization than the conventional MBN. For MI detection, the EvoMBN achieves an overall accuracy of 90.80% and an F1 score of 0.936, whereas the overall accuracy and F1 score of the conventional MBN are 88.70% and 0.919, respectively. Furthermore, the EvoMBN obtains an overall accuracy of 75.18% and an *F*1 score of 0.546 in the MI localization. As for the conventional MBN, it achieves an accuracy of 70.79% and an *F*1 score of 0.530 in the MI localization. To summarize, the architecture learned from the PTB database still has advantages in the transferring experiments compared with the conventional MBN. It demonstrates the superior generalization of our EvoMBN.

### 4.3. Comparison with the State-of-the-Art Models

In this part, the proposed EvoMBN is compared with the other state-of-the-art methods for MI diagnosis using ECGs as listed in Table 7. Note that only the methods evaluated under the inter-patient paradigm are employed during the comparison. 

For the methods using conventional machine learning [16,54] should extract multiple hand-designed features to implement the MI diagnosis. In addition, they only perform MI detection on the PTB database. Considering their results for MI detection, the overall accuracies are only 81.71% and 92.69%, respectively. All the models in [24,27,28] employ the conventional MBN skeleton to implement the MI diagnosis without hand-designed feature extraction. The ML-Net in [24] achieves the best performance for MI detection and localization, according to the experimental results. However, the ML-Net concentrates on the detection and localization of GAMI, which only includes AMI, ASMI, and ALMI. Moreover, the MFB-CBRNNs in [27] are only evaluated by the MI detection experiments. The overall accuracy is less than 95%, whereas all the other MBN models can achieve better performances on MI detection (*Acc* > 95%). The ML-ResNet implements a more comprehensive MI diagnosis in [28]. For MI detection, it obtains an accuracy of 95.49% and an *F*1 score of 0.969. For MI localization, the accuracy and *F*1 score are 55.74% and 0.479, respectively. Note that, the ML-ResNet utilizes all five MI subcategories mentioned in this paper, but the performance still needs to be improved. In [43], a multi-lead attention model is proposed to detect and localize MIs. Using the five aforementioned MI subcategories, it demonstrates better generalization than the ML-ResNet, especially in the MI localization. The accuracies of MI detection and localization are 96.50% and 62.94%, respectively. 

All the aforementioned studies has been listed in Table 7. Considering all the aspects in Table 7, our EvoMBN shows significant advantages over the other methods. First, it is a DL-model using the MBN skeleton, thus, no explicit feature engineering is required. Second, it employs a GA to automatically optimize the architecture to achieve a more accurate MI diagnosis. The efficient LSE mechanism can also improve the model generalization. Furthermore, it achieves a promising performance in the experiments and outperforms the other existing methods. On the PTB database, the overall accuracy and F1 score of MI detection are 97.11% and 0.983, respectively. For MI localization, the model obtains an accuracy of 71.65% and an *F*1 score of 0.694. To the best of our knowledge, the EvoMBN may be the first MI diagnosis model that is evaluated by the architecture transferring experiments. In detail, the accuracies of MI detection and localization are 90.80% and 75.18%, respectively. These superior results indicate the efficiency of the proposed method.

## 5. Conclusions

To overcome the limitations of the conventional MBNs, this paper develops an EvoMBN for MI diagnosis using ECGs. Using a novel fixed-length encoding method, it employs a GA to automatically optimize the architecture, which can be represented by an individual in a population. The operators are designed to implement the evolutional iterations, including crossover, mutation, selection, and fitness evaluation. In addition, a novel LSE mechanism is proposed to emphasize the critical leads for a specific MI subcategory. The model is evaluated under the inter-patient paradigm. Five-fold cross validation is performed on the PTB database. The GA optimization and LSE mechanism have shown superior efficiency in both MI detection and localization. The generalization of the model has been further verified by the architecture transferring experiment on the PTB-XL database. Therefore, the EvoMBN has the potential to assist in MI diagnosis in real-world applications as it shows good performance in all the experiments. In the future, the proposed model will be extended to the diagnosis of other CVDs. Moreover, the GA applied to the MBN should be further explored and improved to achieve better results, especially for MI localization.

## Figures and Tables

**Table 1:** The performance of MI localization using a single multi-class classifier on the PTB database.

Class	*Acc* (%)	*Sen* (%)	*Spe* (%)	*Ppv* (%)	*F*1
HC	59.21	88.84	92.41	73.07	0.802
AMI		39.37	91.43	37.19	0.382
ASMI		59.97	89.37	59.26	0.596
ALMI		42.68	91.31	40.15	0.414
IMI		48.85	91.34	62.36	0.548
ILMI		65.27	95.02	69.02	0.671
Mean	59.21	57.50	91.81	56.84	0.569

**Table 2:** The performance of MI localization using a group of binary classifiers on the PTB database.

Class	*Acc* (%)	*Sen* (%)	*Spe* (%)	*Ppv* (%)	*F*1
HC	71.65	88.21	97.48	89.02	0.886
AMI		42.10	95.60	55.23	0.478
ASMI		70.49	89.81	64.09	0.671
ALMI		66.09	91.71	52.32	0.584
IMI		70.55	96.24	84.65	0.770
ILMI		81.38	95.13	73.98	0.775
Mean	71.65	69.80	94.34	69.88	0.694

**Table 3:** The related anatomical area of each lead.

Aspect	Leads
Anterior	V3, V4
Septal	V1, V2
Lateral	I, aVL, V5, V6
Inferior	II, III, aVF
Endocardial	aVR

**Table 4:** The architectures and performances of the best fold in the five-fold cross validation.

Class	Individual	*Acc* (%)	*Sen* (%)	*Spe* (%)	*Ppv* (%)	*F*1
HC	[17,12,17,2,2,16, 14,2,2,14,16,17]	79.42	93.59	98.19	96.26	0.949
AMI	[10,2,6,6,2,12, 2,6,16,2,17,2]		39.41	94.29	47.09	0.429
ASMI	[8,2,6,10,6,12, 12,2,16,4,6,4]		76.77	91.28	55.53	0.644
ALMI	[14,14,8,8,12,8,17, 17,17,16,14,10]		80.81	96.59	71.75	0.760
IMI	[16,6,16,17,2,12 ,4,2,17,6,2,16]		83.94	96.98	88.59	0.862
ILMI	[17,14,12,8,10,8, 16,14,8,4,10,4]		71.22	98.59	86.71	0.782
**Mean**	**--**	**79.42**	**74.29**	**95.99**	**74.32**	**0.738**

**Table 5:** The MI detection results on the PTB-XL database.

Model	*Acc* (%)	*Sen* (%)	*Spe* (%)	*Ppv* (%)	*F*1
MBN	88.70	87.02	93.31	97.27	0.919
EvoMBN	90.80	92.59	85.88	94.73	0.936

**Table 6:** The MI localization results on the PTB-XL database.

Model	Class	*Acc* (%)	*Sen* (%)	*Spe* (%)	*Ppv* (%)	*F*1
MBN	HC	70.79	94.75	87.08	72.79	0.823
	AMI		27.95	97.72	38.35	0.323
	ASMI		94.43	79.21	70.94	0.810
	ALMI		18.18	99.80	63.41	0.283
	IMI		30.60	99.28	94.27	0.462
	ILMI		59.23	96.53	40.28	0.480
	**Mean**	**70.79**	**54.19**	**93.27**	**63.34**	**0.530**
EvoMBN	HC	75.18	88.21	92.34	80.77	0.843
	AMI		35.34	95.66	29.25	0.320
	ASMI		83.02	92.39	85.42	0.842
	ALMI		22.37	97.37	14.10	0.173
	IMI		69.35	91.16	75.12	0.721
	ILMI		31.01	98.80	50.57	0.384
	**Mean**	**75.18**	**54.88**	**94.62**	**55.87**	**0.547**

**Table 7:** Comparison between existing methods and ours on MI Diagnosis using ECGs.

Method	Hand-Designed Features	Results	
[54] (2018)	10	Detection(IMI): *Sen* = 79.01%; *Spe* = 79.26%; *Ppv* = 80.25%; *Acc* = 81.71%	Localization: NA
[16] (2019)	22	Detection: *Sen* = 80.96%; *Ppv* = 86.14%; *Acc* = 92.69%	Localization: NA
[27] (2020)	0	Detection: *Sen* = 94.42%; *Spe* = 86.29%; *Acc* = 93.08%	Localization: NA
[43] (2020)	0	Detection: *Sen* = 97.10%; *Spe* = 93.34%; *Acc* = 96.50%	Localization: *Sen* = 63.97%; *Spe* = 63.00%; *Acc* = 62.94%
[24] (2021)	0	Detection(GAMI): *Sen* = 94.30%; *Spe* = 97.72%; *Acc* = 96.65%	Localization(GAMI): *Sen* = 62.64%; *Spe* = 68.70%; *Acc* = 66.85%
[28] (2021)	0	Detection: *Sen* = 94.85%; *Spe* = 97.37%; *Acc* = 95.49% *F*1 = 0.969	Localization: *Sen* = 47.58%; *Spe* = 55.37%; *Acc* = 55.74% *F*1 = 0.479
**Proposed ^1^**	**0**	**Detection:** ***Sen* = 98.53%; *Spe* = 90.02%; *Ppv* = 98.01%** ***Acc* = 97.11%; *F*1 = 0.983**	**Localization:** ***Sen* = 69.80%; *Spe* = 94.34%; *Ppv* = 69.88%** ***Acc* = 71.65%; *F*1 = 0.694**
**Proposed ^2^**	**0**	**Detection:** ***Sen* = 92.59%; *Spe* = 85.88%; *Ppv* = 94.73%** ***Acc* = 90.80%; *F*1 = 0.936**	**Localization:** ***Sen* = 54.88%; *Spe* = 94.62%; *Ppv* = 55.87%** ***Acc* = 75.18%; *F*1 = 0.546**

**Proposed ^1^:** The model developed on the PTB database. **Proposed ^2^:** The model transferred to the PTB-XL database.

## Data Availability

PTB and PTB-XL database are available at https://www.physionet.org/content/ptbdb/1.0.0/ and https://www.physionet.org/content/ptb-xl/1.0.1/, respectively (accessed on 15 November 2021).
